# Genetic Characterization of the Fish *Piaractus brachypomus* by Microsatellites Derived from Transcriptome Sequencing

**DOI:** 10.3389/fgene.2018.00046

**Published:** 2018-02-22

**Authors:** Paulo H. Jorge, Vito A. Mastrochirico-Filho, Milene E. Hata, Natália J. Mendes, Raquel B. Ariede, Milena Vieira de Freitas, Manuel Vera, Fábio Porto-Foresti, Diogo T. Hashimoto

**Affiliations:** ^1^Aquaculture Center of Universidade Estadual Paulista Júlio de Mesquita Filho, São Paulo State University, Jaboticabal, Brazil; ^2^Veterinary Faculty, University of Santiago de Compostela, Lugo, Spain; ^3^School of Sciences, São Paulo State University, Bauru, Brazil

**Keywords:** aquaculture, genetic structure, NGS, Pirapitinga, Serrasalmidae

## Abstract

The pirapitinga, *Piaractus brachypomus* (Characiformes, Serrasalmidae), is a fish from the Amazon basin and is considered to be one of the main native species used in aquaculture production in South America. The objectives of this study were: (1) to perform liver transcriptome sequencing of pirapitinga through NGS and then validate a set of microsatellite markers for this species; and (2) to use polymorphic microsatellites for analysis of genetic variability in farmed stocks. The transcriptome sequencing was carried out through the Roche/454 technology, which resulted in 3,696 non-redundant contigs. Of this total, 2,568 contigs had similarity in the non-redundant (nr) protein database (Genbank) and 2,075 sequences were characterized in the categories of Gene Ontology (GO). After the validation process of 30 microsatellite loci, eight markers showed polymorphism. The analysis of these polymorphic markers in farmed stocks revealed that fish farms from North Brazil had a higher genetic diversity than fish farms from Southeast Brazil. AMOVA demonstrated that the highest proportion of variation was presented within the populations. However, when comparing different groups (1: Wild; 2: North fish farms; 3: Southeast fish farms), a considerable variation between the groups was observed. The *F*_ST_ values showed the occurrence of genetic structure among the broodstocks from different regions of Brazil. The transcriptome sequencing in pirapitinga provided important genetic resources for biological studies in this non-model species, and microsatellite data can be used as the framework for the genetic management of breeding stocks in Brazil, which might provide a basis for a genetic pre-breeding programme.

## Introduction

The pirapitinga (*Piaractus brachypomus*) is a native fish from the Amazon and Orinoco Rivers and can reach up to 20 kg of weight (Alcântara et al., 1990). This species is used for fish farming, is valued for its meat and has fast growth performance (Fresneda et al., 2004). In Brazil, pirapitinga farming represents the third largest fish production operation (about 10,000 tons) among the native fish species (MPA, 2013a). Furthermore, this species has been widely used for the production of interspecific hybrids, particularly the tambatinga (female tambaqui *Colossoma macropomum* × male pirapitinga *P. brachypomus*), and patinga (female pacu *Piaractus mesopotamicus* × male pirapitinga *P. brachypomus;* IBGE, 2016). The aquaculture production of pirapitinga in Brazil is concentrated mainly in the Midwest and North (87%), followed by the Southeast (9%), Northeast (3%) and South (1%) (MPA, 2013b). This species also has economic importance for aquaculture in other countries in South America (Colombia, Peru, and Venezuela) and in Asia (China, Myanmar, Thailand, and Vietnam; Flores Nava, 2007; Honglang, 2007; Lin et al., 2015).

However, despite this representation of aquaculture production, few scientific studies have focused on understanding the biology of pirapitinga, especially of genetic traits. So, the generation of genetic resources for this species is fundamental to advancing studies of breeding and genetic management, as occurred in model species used in aquaculture, such as salmon, catfish, carp, and tilapia (Lien et al., 2011; Liu et al., 2011; Guyon et al., 2012; Ji et al., 2012).

Model fish species, such as zebrafish *Danio rerio*, have been described with more than 26,000 genes (Howe et al., 2013). However, few genes and their metabolic pathways have been characterized for non-model species without reference genomes, as is the case for pirapitinga. In the field of genetics and molecular biology, Next-Generation Sequencing (NGS) technologies are causing a revolution, allowing the sequencing of genome and transcriptome of any organism, quickly and at low cost (Seeb et al., 2011). RNA-seq (transcriptome sequencing) is considered one of the most used strategies of NGS technology for the transcripts analysis (Qian et al., 2014), wherein all the messenger RNA (mRNA) of a specific tissue or set of tissues are used as a source for sequencing. Moreover, RNA-seq is an effective tool for discovery of molecular markers, particularly for the prospection of gene-associated microsatellites (Teacher et al., 2012; Xu et al., 2013).

Due to the usefulness of revealing the genetic variation among individuals (Liu and Cordes, 2004), microsatellite markers have proven to be efficient for genetic characterization of wild populations and breeding stocks of farmed fish (Koljonen et al., 2002; Lehoczky et al., 2005), such as to prevent inbreeding (Ponzoni et al., 2008), to identify and preserve live gene banks (Machado-Schiaffino et al., 2007), to detect genetic structure (Do Prado et al., 2018), to direct matings during the formation of the population base of breeding programmes (Fernández et al., 2014), and to perform marker assisted selection (MAS) for economic traits (Houston et al., 2010). However, these markers are not available for pirapitinga, one of most important species for the aquaculture in South America.

For the aquaculture of pirapitinga, analysis of genetic variability in farmed stocks still needs to be performed, which will allow three hypotheses to be tested: (1) farmed stocks of pirapitinga have lower genetic diversity in relation to wild stocks; (2) farmed stocks of pirapitinga in Brazil are genetically structured; and (3) gene-linked microsatellites can be associated to economic traits of pirapitinga, such as growth and disease resistance. These analyses will support the creation of a breeding programme to increase the productivity of pirapitinga, by directed matings which lead to the formation of families, avoiding the problems of bottlenecks and inbreeding in the base population (Fernández et al., 2014), and by the identification of quantitative trait loci (QTL), which will assist the selection of superior genotypes by MAS (Houston et al., 2010).

Thus, the objective of the present study was to characterize genetic resources for the proper management of this non-model species in aquaculture, through transcriptome characterization and genetic variability analysis of stocks using microsatellite markers.

## Materials and methods

### Ethics statement

This study was carried out in strict accordance with the animal welfare guidelines of the National Council for Control of Animal Experimentation (Brazilian Ministry for Science, Technology, and Innovation). The present study was performed under authorization N° 33435-1, issued through ICMBio (Chico Mendes Institute for the Conservation of Biodiversity, Brazilian Ministry for Environment). No animal was housed or cared for in the laboratory. Fish were euthanized by benzocaine anesthetic overdose for collection of liver tissue for transcriptome sequencing. For microsatellite validation and genetic variability analysis, fin fragments were collected from each fish under benzocaine anesthesia and all efforts were made to minimize suffering.

### Samples for transcriptome sequencing

To perform the transcriptome sequencing, samples of liver tissue were taken from 10 individual fish from three different Brazilian fish farms and one wild population: Aquaculture Center of São Paulo State University, CAUNESP, Jaboticabal, SP (*n* = 3); Projeto Peixe fish farm, Sales Oliveira, SP (*n* = 1); Fazenda São Paulo fish farm, Brejinho de Nazaré, TO (*n* = 5); and Tocantins River, Lajeado, TO (*n* = 1). Individuals from different origins were used in order to achieve the highest genetic variability in microsatellite discovery analysis. Liver samples were selected for transcriptome studies because the liver plays a critical role in coordinating various physiological processes, including digestion, metabolism, detoxification, and endocrine system immune response (Martin et al., 2010).

### Samples for genetic variability analysis

Analyses of microsatellite validation were performed in 22 individual pirapitinga collected from the Tocantins River (TO) from Lajeado City, Tocantins State, Brazil. We then used the microsatellite markers to study the genetic variability in samples collected from four commercial fish farms: TO1 (*n* = 25) and TO2 (*n* = 26), from Tocantins State (North Brazil); and SP1 (*n* = 36) and SP2 (*n* = 20), from São Paulo State (Southeast Brazil). To maintain the confidentiality of these fish farms, the names of and information on the fish farms have been preserved.

### Analysis of genetic purity in pirapitinga individuals

According Hashimoto et al. (2014), interspecific hybrids have been detected in broodstocks of Brazilian fish farms. The pirapitinga can be crossed with tambaqui *C. macropomum* or pacu *P. mesopotamicus*, resulting in viable and fertile hybrids (Hashimoto et al., 2012, 2014). Therefore, in the present study, special attention was given to analyze pure pirapitinga, and not interspecific hybrids. The analysis of genetic purity in all animals herein studied was performed using the mitochondrial genes, Cytochrome C Oxidase subunit I (*mt-co1*) and Cytochrome b (*mt-cyb*); and the nuclear genes, α-Tropomyosin (*tpm1*) and Recombination Activating Gene 2 (*rag2*), according to the protocols and methods of Hashimoto et al. (2011). Fish identified as interspecific hybrids were excluded from further analysis in this study.

### cDNA library construction and Roche 454 platform sequencing

Samples of ~100 mg of liver fixed in RNAlater were extracted with Rneasy Mini Kit (Qiagen). Each sample was quantified by spectrophotometry using NanoDrop ND-1000 equipment and the quality (integrity) was checked by 2100 Bioanalyzer equipment. It succeeded the preparation of an equimolar pool of total RNA samples (from 10 individuals) to mRNA enrichment with μMACS mRNA Isolation Kit (Miltenyi Biotech).

A non-normalized cDNA library was prepared using cDNA Synthesis System Kit with random primer GS Rapid Library Prep Kit and GS Rapid Library MID Adaptors Kit (Roche). The High Sensitivity DNA LabChip Kit (Agilent Technologies) with 2100 Bioanalyzer was used for quality analysis of the cDNA library. The concentration of sample (molecules/μL) was obtained by QuantiFluor^TM^—ST fluorimeter (Promega). Titration of emPCR (emulsion PCR) was performed with the GS FLX Titanium SV em PCR Kit (Lib-L) (Roche), according to the emPCR Amplification Method Manual—Libl SV, GS FLX+ Series, to identify the optimal number of DNA molecules per bead (cpb = copies per bead). After emPCR titration, the emPCR was performed with GS FLX Titanium LV emPCR Kit (Lib-L) (Roche), according to the emPCR Amplification Method Manual—LibL LV, GS FLX+ Series. The transcriptome sequencing was conducted using the Roche/454 technology (GS FLX Titanium Sequencing Kit XL +) from HELIXXA company (Campinas, SP, Brazil), which has been used for transcriptome analysis of non-model fish species (Renaut et al., 2010).

### Bioinformatic analysis

Filtering of the initial quality of the 454 sequences in sff format was performed using the Roche Newbler programme. Sequence analysis was performed using the high-throughput sequencing module of CLC Genomics Workbench (version 7.5.1; CLC bio, Aarhus, Denmark). The raw reads were cleaned by trimming low quality sequences with quality scores of <20. Terminal nucleotides (five nucleotides at each extremity 5′ and 3′), ambiguous nucleotides, adapter sequences and reads <15 base pairs (bp) were discarded. For *de novo* assembly, contigs <200 bp were also discarded and the default local alignment settings were used to rank potential matches (mismatch cost of 2, insertion cost of 3, deletion cost of 3). The highest scoring matches that shared ≥50% of their length with ≥80% of similarity were included in the alignment. The assembled transcripts were subjected to cd-hit-est programme with an identity threshold of 90% to remove redundancy (Li and Godzik, 2006; Duan et al., 2012). In order to remove any mitochondrial and ribosomal contamination, sequences were compared against pacu mitochondrial genome and zebrafish ribosomal RNA RefSeqs (NCBI database) using CLC Genomic Workbench (version 8.0.3; CLC Bio, Aarhus, Denmark).

Functional annotation of the unique consensus sequences was performed by homology searches against the *National Center for Biotechnology Information* (NCBI) non-redundant protein database (nr) (cutoff E-value of 1E-3) using BLAST2GO software (Conesa et al., 2005) to obtain the putative gene identity. All BLASTx hits were filtered for redundancy in protein accessions. The gene ontology (GO) terms were assigned to each unique gene based on the GO terms annotated to the corresponding homologs in the NCBI database (e-value cutoff 1e-6). The transcripts were further annotated in InterPro, Enzyme code (EC), and Kyoto Encyclopedia of Genes and Genomes (KEGG) metabolic pathways analysis through the Bi-directional Best Hits (BBH) method.

Microsatellites were identified in the contigs using msatcommander software (Faircloth, 2008). Primers flanking the microsatellite loci were designed with Primer3plus software (Rozen and Skaletsky, 2000). The six possible reading frames of the consensus sequence of each functionally annotated contig containing microsatellite were compared against the NCBI protein database using BLASTx (e-value 1e-10) in order to find *Open Reading Frame* (ORF) regions. These approaches allowed us to locate microsatellites in coding sequences (CDS) or untranslated regions (5′UTR and 3′UTR) through graphical sequence viewer Tablet (Milne et al., 2013).

### Microsatellite genotyping and validation

DNA was extracted from fin fragments using the Wizard Genomic DNA Purification Kit (Promega), according to the manufacturer's protocol. Microsatellite validation was performed in 30 loci, selected according to the motif and functional annotation of the contigs. Amplifications were performed by polymerase chain reaction (PCR) in a total volume of 25 μl containing 100 μM of each dNTP (dATP, dTTP, dGTP, and dCTP), 1.5 mM MgCl_2_, 1X *Taq* DNA buffer (20 mM Tris-HCl, pH 8.4, and 50 mM KCl), 0.1 μM of each primer, 0.5 units of *Taq* Polymerase (Invitrogen) and 10-50 ng of genomic DNA. The reactions were performed in a thermocycler (ProFlex™ PCR System, Life Technologies) following initial denaturing for 10 min at 95°C; 35 cycles of 30 s at 95°C, 30 s at 55–60°C (adjusted for each primer set), 20 s at 72°C; and a final extension at 72°C for 20 min.

Microsatellites that showed polymorphism in 6% polyacrylamide gels were analyzed in a 3130xl sequencer (Life Technologies) to get better accuracy of allele determination. The sequencing strategy adopted in this study was according to protocols described by Schuelke (2000), using the CAGtag primer (5′-CAGTCGGGCGTCATCA-3′; Shirk et al., 2013) labeled with the fluorochromes HEX or FAM. The genotyping PCR was performed with the following reagents: 100 μM of each dNTP, 1.5 mM MgCl_2_, 1X *Taq* DNA buffer, 0.1 μM of each primer (F and R), 0.01 μM of the CAGtag primer, 0.5 units of *Taq* Polymerase (Invitrogen), and 10–50 ng of genomic DNA. The cycling programme for amplification consisted of: nine cycles at 95°C for 30 s, 55–60°C for 30 s (adjusted for each primer set), 72°C for 20 s; then, 30 cycles at 95°C for 30 s, 50°C for 30 s, and 72°C for 20 s. During the first nine cycles, the annealing temperature of 55–60°C allows incorporation of the primers (F and R) from the microsatellite loci. Then, in the following 30 cycles, the temperature of 50°C facilitates the annealing of the fluorescent dye-labeled CAGtag primer. PCR products were analyzed by capillary electrophoresis with a 3130xl genetic analyzer, using the DS-30 matrix, with the GeneScan 500 ROX dye Size Standard (Thermo). The programme GeneMapper 3.7 (Applied Biosystems) was used to determine the allele sizes.

### Microsatellite diversity and population analysis

For statistical analysis, we initially used GenAlex analysis 6.1 software (Peakall and Smouse, 2012) to convert the arrays into specific formats for each programme. The observed (*H*_*o*_) and expected (*H*_*e*_) heterozygosity, Hardy-Weinberg Equilibrium (HWE) and Analysis of Molecular Variance (AMOVA) (Excoffier et al., 1992) were calculated using the Arlequim 3.5 programme (Excoffier and Lischer, 2010). The levels of significance for the HWE test were adjusted with the Bonferroni correction (Rice, 1989). The inbreeding coefficient (*F*_IS_) was performed using Genepop 4.0.11 (Rousset, 2008), based on Weir and Cockerham (1984) estimates. The fixation index (*F*_ST_) was calculated using FSTAT 9.3.2 software (Goudet, 1995). Wright (1965) threshold values were adopted, *F*_ST_ = little genetic differentiation (0–0.05); moderate genetic differentiation (0.05–0.25); high level of genetic differentiation (> 0.25). The programme Cervus v.3.0.7 (Marshall et al., 1998) was applied to verify the presence of null alleles. Linkage disequilibrium (LD) was estimated using Arlequin v.3.5.2.2. The levels of significance were adjusted to multiple tests using the Bonferroni correction.

After LD analysis, level of admixture among population samples was inferred by estimating the optimum number of clusters (K), as suggested by Evanno et al. (2005), using the programme STRUCTURE version 2.3.4 (Pritchard et al., 2000) without prior information about population. Primarily, we determined the distribution of ΔK, an *ad hoc* statistic based on the rate of change in the log probability of data between successive K values. The range of clusters (K) was predefined from 1 to 5. The analysis was performed in 25 replicated runs using 200,000 iterations after a burn-in period of 50,000 runs. The K value most likely to explain the population structure is the modal value of this ΔK. The outputs of STRUCTURE analysis were visualized through the STRUCTURE HARVESTER programme (Earl, 2012).

Analysis for population bottlenecks was tested using BOTTLENECK (Cornuet and Luikart, 1996; Piry et al., 1999), by using the mutation–drift equilibrium assuming the two-phase model (TPM) with 70% stepwise mutation model (SMM) and 30% infinite allele model (IAM). Deviations between the observed and expected frequency distributions were tested using the Wilcoxon's signed rank test. BOTTLENECK was run for 10,000 iterations.

## Results

### Transcriptome sequencing

The results of liver transcriptome sequencing in pirapitinga yielded a total of 192,373 reads, which were deposited in the Short Read Archive (SRA) of NCBI under the accession number SRR6303971. The raw reads presented an average length of 395.5 bp, comprising a total of ~76 Mbp. After the trimming process, the average length of the reads was of 362.1 bp, resulting in a total of ~69 Mbp (192,077 *reads;* Table 1). As *P. brachypomus* is considered a non-model organism, and therefore without reference genome, *de novo* assembly strategy was performed for transcriptome analysis, which yielded 3,696 non-redundant contigs as a result of 174,272 overlapping reads (63,460,229 bp). The size characteristics of the contigs are presented in Table 1. A total of 17,805 remaining reads (6,084,530 bp) was considered as singletons, and therefore they were not used for subsequent analysis.

**Table 1:** Data of *de novo* assembly from liver transcriptome of pirapitinga *Piaractus brachypomus*.

Matched reads for assembly	174,272
Total nucleotides of matched reads	63,460,229
Number of contigs	3,797
Total of contig nucleotides	2,999,680
Minimum contig length (bp)	202
Maximum contig length (bp)	7,812
Average contig length (bp)	790
N75 (bp)	593
N50 (bp)	861
N25 (bp)	1,383

Non-redundant sequences were annotated by BLASTx algorithm against the NCBI databases: non-redundant protein (*nr*), protein RefSeq of *zebrafish* and *fugu*. A total of 2,568 unique protein accessions (69.4% of transcripts) had significant similarity in the *nr* database. In relation to the protein RefSeq of *zebrafish* and *fugu*, we found similar numbers of annotated genes, which were of 2,498 (67.6%) and 2,419 (65.4%), respectively. No sequence showed homology with known pirapitinga protein sequences deposited in NCBI database, because the available sequences database is still limited to mostly mitochondrial sequences.

**Figure 1:**
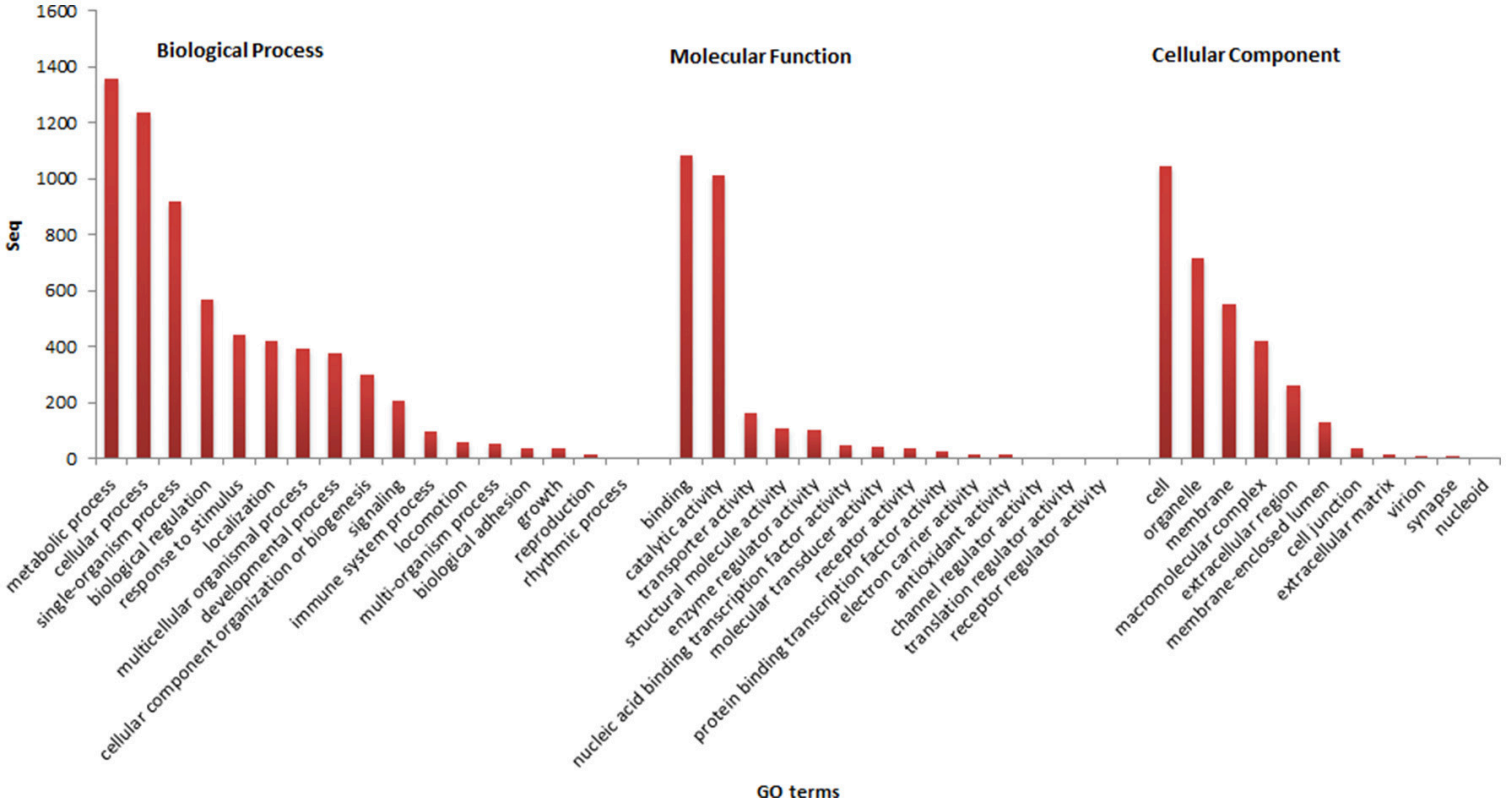
Results of functional annotation and the assignment of genes in the GO categories and subcategories.

Of the 2,568 contigs with correspondence in the *nr* database, 2,075 (80.8%) were annotated in the categories of Gene Ontology (GO). A total of 1,831 assignments to Biological Process (88.2%) were found, followed by 1,757 to Molecular Function (84.6%) and 1,378 to Cellular Component (66.4%). In relation to the GO subcategories, the most abundant terms were related to: metabolic process, cellular process, and single-organism process of the Biological Process category; binding, catalytic activity, and transporter activity of the Molecular Function; cell, organelle, and membrane of the Cellular Component (Figure 1). In the present study, genes assigned to the immune system, growth and reproduction were found, and therefore these data will serve as support for future studies on the aquaculture of pirapitinga.

**Figure 2:**
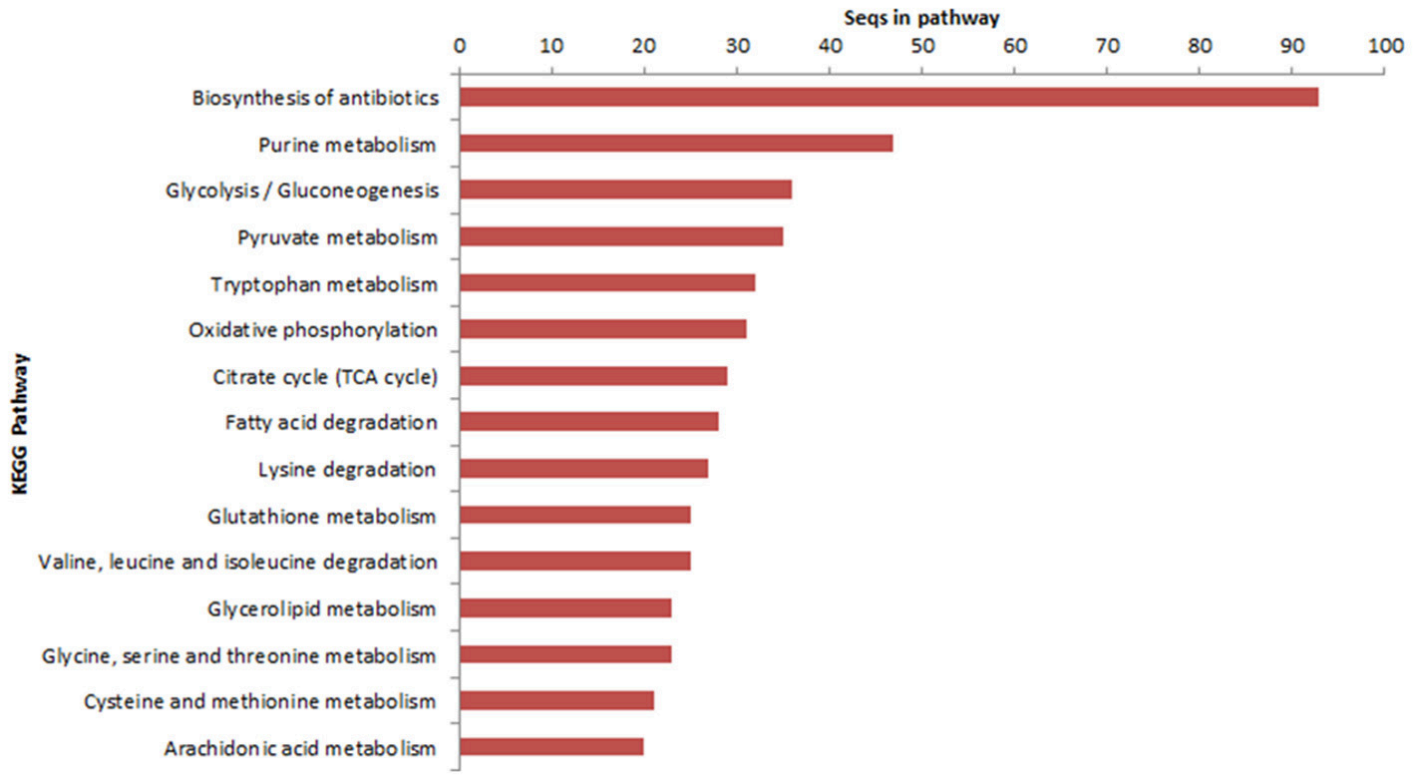
Transcripts characterized in metabolic pathways database of KEGG enzymes *(Kyoto Encyclopedia of Genes and Genomes*).

The transcripts characterization in the KEGG database demonstrated that 1,122 sequences were identified in 106 metabolic pathways. Genes involved in the biosynthesis of antibiotics, purine metabolism and glycolysis/gluconeogenesis were able to be highlighted (Figure 2).

### Microsatellite diversity and population analysis

The search for short sequence repeats (SSR) in the 3,696 contigs resulted in the discovery of 130 microsatellite markers distributed in 95 contigs. In total, 75 pairs of primers were designed adjacent to the microsatellite loci, including the following sequence repeats: 56 di, 13 tri, 4 tetra, and 2 pentanucleotide. Among the dinucleotide motifs, the main repeats were the types AC (48.28%), AG (39.65%), AT (10.35%), and CG (1.72%). In relation to the trinucleotide motifs, we identified seven types (AGC, AGG, ATC, AAT, ACG, CCG, and AAG). The tetranucleotide (ATCT, AAAG, AATG, and AAAT) and pentanucleotide (ACTAT and ATAGT) sequences were described with the presence of four and two types of motifs. In relation to the gene position, 26.76% of the microsatellite markers were found in the 3′UTR (untranslated region), 19.71% in the 5′UTR, and 29.58% in the cds (coding sequence).

In the process of microsatellite validation, 30 markers were evaluated in 22 samples of pirapitinga collected from the wild. Of these markers, eight microsatellite loci showed polymorphism (GenBank accession numbers MG595996—MG596003), revealed by the presence of different fragment sizes (Table 2). The number of alleles was low, which ranged from 2 (loci C25, C64, C410, and C1832) to 5 (C1376) and mean of 2.750 ± 0.366. The expected (*H*_*e*_) and observed (*H*_*o*_) heterozygosity in the wild population had an average of 0.466 ± 0.061 and 0.355 ± 0.076, respectively. Most of the loci showed positive values for *F*_IS_, except the locus C64. Three microsatellite loci (C13, C25, and C1716) showed significant deviation from the Hardy–Weinberg Equilibrium (HWE) after Bonferroni correction (adjusted *p* = 0.00625).

**Table 2:** Characterization of the genetic diversity of eight polymorphic microsatellites in the wild population of pirapitinga (*Piaractus brachypomus*).

**Locus**	**Sequence description**	**Gene position**	**Motifs**	**Primers 5′−3′**	**${\text{T}}_{{\text{A}}^{{}^{\circ}}}$C**	**Size range**	**Na**	**H_o_**	**H_e_**	**P(HWE)**	***F*_IS_**	**F(Null)**
C13	dihydroxyvitamin d24-like	3′UTR	(AGC)_6_	F: TCTCTTCAAGCCTCCTCTGC R: ATGCTGCAGCTCCTCCTGT	60°C	143–149	3	0.545	0.669	0.000	0.188	0.075
C25	cytosolic 5-nucleotidase 3a-like isoform x 1	3′UTR	(AT)_11_	F: CTTTGTCTGCTTTGGGTCGT R: CTTAGAAGAATGTGCAAATTGAAA	60°C	117–120	2	0.000	0.169	0.001	1.000	0.887
C64	sodium-coupled neutral amino acid transporter	5′UTR	(AAAG)_7_	F: CAAAGCAAACTCAAAAAGGAAAA R: TGGGAACGTTTAGCATCTCA	55°C	143–151	2	0.545	0.474	0.650	−0.156	−0.082
C410	apolipoprotein e	3′UTR	(AG)_8_	F: CGCACAGGTCTAAAGGCACT R: CTCCCACACAGTGAAAAGCA	60°C	125−127	2	0.273	0.359	0.271	0.245	0.125
C1005	apolipoprotein e	3′UTR	(AG)_7_	F: AGTTGTTGCACCAAATGCAG R: CTTGTTCCCTCCCACACAGT	60°C	137−141	3	0.318	0.369	0.225	0.140	0.090
C1376	frutose-biphosphatase 1-like	5′UTR	(AC)_10_	F: GTGTTACATGGCAGGCGTTT R: CAAGTGAGACCAAATCCAAGG	60°C	157–175	5	0.500	0.608	0.185	0.180	0.073
C1716	vacuolar atp synthase 16 kda proteolipid subunit	3′UTR	(GT)_7_	F: AACCGAAGAGAGGGGAGTGT R: GCATTTACAAGGGGACGCAC	60°C	155–171	3	0.545	0.659	0.000	0.175	0.059
C1832	–	–	(AC)_6_	F: GGTGCTATGTCGTAGAGGCC R: AGGAAGGCATGACCAGTGTG	60°C	159–169	2	0.111	0.529	0.036	0.800	ND

*The wild population analyzed corresponds to 22 individuals collected on the Tocantins River. T_A_, annealing temperature (°C); Na, number of alleles per locus; H_o_, observed heterozygosity; H_e_, expected heterozygosity; P (HWE), p-value from Hardy-Weinberg equilibrium and F_IS_, inbreeding coefficient. F(Null), Null alleles; ND, not performed*.

The results of genetic variability in farmed stocks revealed that North fish farms TO1 and TO2 had higher diversity than the wild population, demonstrated by number of alleles (mean of 4.500 ± 0.423 and 3.375 ± 0.596, respectively) and average values of *H*_*e*_ (0.589 ± 0.033 and 0.488 ± 0.044, respectively) and *H*_*o*_ (0.520 ± 0.060 and 0.447 ± 0.040, respectively; Table 3). The Southeast fish farms SP1 and SP2 showed the lowest genetic variability when compared to other populations, with lower allele number (mean of 2.250 ± 0.313 and 3.125 ± 0.581, respectively), and average of *H*_*e*_(0.226 ± 0.077 and 0.278 ± 0.085, respectively; *p* < 0.05) and *H*_*o*_ (0.259 ± 0.103 and 0.251 ± 0.079, respectively; Table 3). Most of the microsatellite loci were characterized with positive values of *F*_IS_, except for SP1 and SP2. The mean value of *F*_IS_ and null alleles was positive in most populations, with the exception of SP1 (−0.071 ± 0.076 and −0.011 ± 0.080). The majority of the markers were in concordance to HWE, after Bonferroni correction, with the exception of C25 (TO1, SP1, and SP2), C64 (SP1 and SP2) and C1376 (SP1) (Table 3). Linkage disequilibrium was found between the microsatellites C410 and C1005 (*p* < 0.00625). Although molecular markers on linkage disequilibrium were not applied in genetic variability studies, this information can be useful in future analysis of genetic mapping.

**Table 3:** Values of genetic diversity of eight microsatellite loci of *Piaractus brachypomus*.

**Stocks**		**Loci**							
		**C13**	**C25**	**C64**	**C410**	**C1005**	**C1376**	**C1716**	**C1832**
Wild	N	22	22	22	22	22	22	22	9
	Na	3	2	2	2	3	5	3	2
	H_o_	0.545	0.000	0.545	0.273	0.318	0.500	0.545	0.111
	H_e_	0.669	0.169	0.474	0.359	0.369	0.608	0.659	0.529
	P(HWE)	0.000	0.001	0.650	0.271	0.225	0.185	0.000	0.036
	*F*_IS_	0.188	1.000	−0.156	0.245	0.140	0.180	0.175	0.800
	F(Null)	0.075	0.887	−0.082	0.125	0.090	0.073	0.059	ND
TO1	N	25	25	25	25	25	25	25	25
	Na	4	5	3	3	4	6	6	5
	H_o_	0.600	0.160	0.560	0.520	0.560	0.440	0.760	0.560
	H_e_	0.536	0.704	0.495	0.537	0.562	0.609	0.782	0.580
	P(HWE)	0.650	0.001	0.365	0.267	0.421	0.010	0.545	0.388
	*F*_IS_	−0.121	0.776	−0.133	0.034	0.004	0.282	0.028	0.035
	F(Null)	−0.067	0.620	−0.065	−0.015	−0.034	0.164	0.011	−0.001
TO2	N	26	21	26	26	26	26	26	26
	Na	3	2	2	2	2	6	5	5
	H_o_	0.500	0.380	0.346	0.384	0.384	0.538	0.461	0.653
	H_e_	0.528	0.315	0.382	0.506	0.506	0.632	0.515	0.638
	P(HWE)	0.840	1.000	0.626	0.256	0.256	0.020	0.030	0.670
	*F*_IS_	0.055	−0.212	0.096	0.244	0.244	0.151	0.107	−0.253
	F(Null)	0.018	−0.103	0.040	0.127	0.127	0.055	0.067	−0.027
SP1	N	14	14	14	14	14	14	14	14
	Na	2	1	1	3	2	3	3	3
	H_o_	0.143	–	–	0.287	0.500	0.857	0.143	0.143
	H_e_	0.137	–	–	0.264	0.494	0.634	0.140	0.203
	P(HWE)	1.000	–	–	1.000	1.000	0.000	1.000	0.109
	*F*_IS_	−0.040	–	–	−0.083	−0.011	−0.368	−0.019	0.306
	F(Null)	−0.028	–	–	−0.069	−0.023	−0.215	−0.027	0.272
SP2	N	19	19	19	19	19	16	18	19
	Na	2	2	1	3	3	5	3	6
	H_o_	0.368	0.000	–	0.157	0.157	0.625	0.222	0.473
	H_e_	0.308	0.193	–	0.152	0.152	0.790	0.207	0.486
	P(HWE)	1.000	0.002	–	1.000	1.000	0.112	1.000	0.387
	*F*_IS_	−0.200	1.000	–	−0.038	−0.385	0.214	−0.070	0.027
	F(Null)	−0.099	0.916	–	−0.032	−0.032	0.093	−0.051	0.024

*Wild, population from the Tocantins River; TO1 and TO2, fish farms from Tocantins; SP1 and SP2, fish farms from São Paulo, Na, number of alleles; H_o_, observed heterozygosity; H_e_, expected heterozygosity; P (HWE), P-value from Hardy-Weinberg equilibrium; F_IS_, inbreeding coefficient; F(Null), Null alleles; ND, not performed*.

In bottleneck analyses, evidence for recent reductions in population size (bottleneck) using TPM was not found, except for the wild population of Tocantins River (*p* = 0.027).

**Figure 3:**
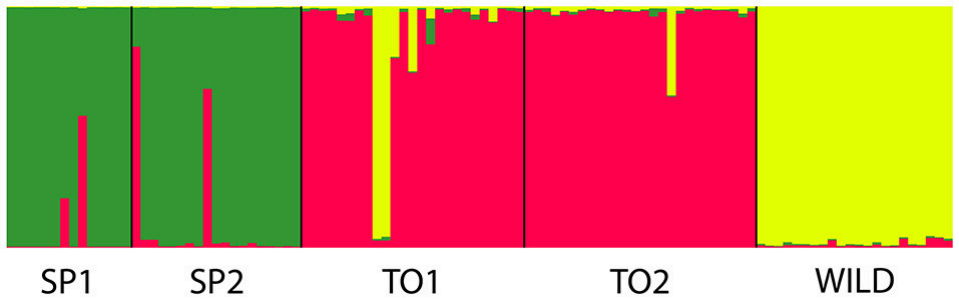
Evaluation of the level of admixture among stocks by STRUCTURE, showing three main clusters between the populations: Group 1 (SP1 and SP2) in green, Group 2 (TO1 and TO2) in red, and Group 3 (wild) in yellow.

In the evaluation of the level of admixture among stocks by STRUCTURE, the model-based clustering analyses detected K = 3, allowing the identification of 3 main clusters between the populations: Group 1 (SP1 and SP2), Group 2 (TO1 and TO2), and Group 3 (wild) (Figure 3).

The global *F*_ST_ was 0.379, which showed high genetic differentiation among the populations (*p* < 0.05). Pairwise *F*_ST_ detected a higher genetic differentiation between the wild population and all farmed stocks, particularly when compared to SP1 (*F*_ST_ = 0.538, *p* < 0.05) and SP2 (*F*_ST_ = 0.537, *p* < 0.05). Additionally, high genetic structure was found between the populations from North and Southeast Brazil, as observed between TO2 with SP1 (*F*_ST_ = 0.463, *p* < 0.05) and TO1 with SP2 (*F*_ST_ = 0.380, *p* < 0.05; Table 4). Moreover, values of pairwise *F*_ST_ after stock clustering detected a higher genetic differentiation when comparing Group 1/Group 2 (*F*_ST_ = 0.379, *p* < 0.05), Group 1/Group 3 (*F*_ST_ = 0.549, *p* < 0.05), and Group 2/Group 3 (*F*_ST_ = 0.144, *p* < 0.05).

**Table 4:** Analysis of pairwise *F*_*ST*_ based on eight microsatellite loci between populations of *Piaractus brachypomus*.

	**SP1**	**SP2**	**TO1**	**TO2**	**wild**
SP1	–				
SP2	0.18438	–			
TO1	0.39201	0.38080	–		
TO2	0.46373	0.42999	0.08345	–	
wild	0.53856	0.53782	0.16086	0.17915	–

*Wild, population from the Tocantins River; TO1 and TO2, fish farms from Tocantins; SP1 and SP2, fish farms from São Paulo. All results of F_ST_ were significant statistically p < 0.05*.

The results of AMOVA showed that the majority of genetic variation (29.11%, *F*_CT_ = 0.291, *p* < 0.001) occurred between groups (according to STRUCTURE clustering), while the variation among individuals within populations was only 8.21% (*F*_IS_ = 0.126, *p* < 0.001) and among populations within groups presented 6.06% of genetic variation (*F*_SC_ = 0.085, *p* < 0.001).

## Discussion

### Transcriptome sequencing

Currently, genetic resources for pirapitinga *P. brachypomus* are limited only to sequences of the mitochondrial genome (Chen et al., 2016). Thus, one of the main results of this study was the data generated through transcriptome sequencing, because little knowledge was available about the genes of this species. The efficiency of the Roche/454 sequencing system in the functional genomics analysis of pirapitinga can be observed because of the 3,696 transcripts that were generated in this study. According to Seeb et al. (2011), genome reduction strategies for NGS sequencing (e.g., transcriptome sequencing) are more viable when the objective is to prospect molecular markers and genetic information for use in aquaculture, in a low cost and fast way. Roche/454 sequencing technology is one of the main methods used in NGS transcriptome of non-model fish (Renaut et al., 2010; Shin et al., 2012; Calduch-Giner et al., 2013; Mutz et al., 2013).

The results of functional annotation showed that the sequences of pirapitinga had a high proportion of annotated genes when compared to the database of *zebrafish* and *fugu* proteins. The gene annotation allowed identification of genomic regions responsible for ontogenetic development processes, biological regulation, the immune system, and regions involved in processes of growth and reproduction. Consequently, the present data can be used as the basis of further biological studies of other areas of aquaculture or for future breeding programmes. In addition, through transcriptome sequencing, the discovery of gene-associated microsatellites can be considered to be the main result which can be applied to pirapitinga aquaculture, as already demonstrated in previous studies of fish (Renaut et al., 2010; Helyar et al., 2012; Shin et al., 2012). The use of gene-associated markers becomes even more important in the construction of genetic maps (Shin et al., 2012) because, by comparative genomics using fish genome references already sequenced, it is possible to presume the location of each studied locus.

Moreover, some examples have demonstrated that gene-linked microsatellite markers can be correlated with interesting productive traits, especially for growth performance. In the fish *Sparus aurata*, a dinucleotide microsatellite in the 5′ UTR of the growth hormone gene (GH) is linked with faster growth rate, especially the alleles 250 and 254, which can be used for breeding management and genetic selection for this trait (Almuly et al., 2005). In other fish species, such as *Oreochromis niloticus* and *Lates calcarifer* (Yue et al., 2001; Yue and Orban, 2002), microsatellites have also been reported for genes of interest (prolactin, GH and igf2) and, therefore, they can be used in marker-assisted selection (MAS) programmes. In the present study, eight polymorphic microsatellite loci were validated, some of them located in gene regions that may be useful for productive characteristics in aquaculture. In this case, a microsatellite *locus* was found in the gene *Tetraspanin*−*3 isoform x1* (C1832), which plays a role in viral infection pathology (Martin et al., 2005; Shoshana and Shoham, 2005). There is another microsatellite in the gene *Cytosolic 5 – nucleotidase 3 a-like* (NTC5C3) (C25), which contributes in the production of red blood cells and its mutation can cause hemolytic anemia and influence on the immune system (Aksoy et al., 2009). Thus, the microsatellites described in this study will be also important in future analysis of (QTL) linked to traits of disease resistance, which has received special attention in aquaculture species, such as turbot (*Scophthalmus maximus*), rainbow trout (*Oncorhynchus mykiss*), salmon (*Salmo salar*), Nile tilapia (*O. niloticus*), and cod (*Gadus morhua*), investigating the resistance to pathogens (Pardo et al., 2008; Ødegård et al., 2010, 2011; Yáñez et al., 2014; Evenhuis et al., 2015). Furthermore, one microsatellite locus was also detected in the gene *apolipoprotein e* (C410), which is associated with the central nervous system and the senescence process (Wang et al., 2014). These markers can provide useful information for studies of the biology of the pirapitinga, besides serving as a framework for other native species.

### Population analysis

The validation of eight microsatellites showed a low level of genetic diversity in these loci, both in wild and farmed stocks. In the wild, the observed heterozygosity (*H*_*o*_) ranged from 0.000 to 0.545 and an average of 2.750 alleles per locus. These values confirm the low genetic variability when compared with related species, such as pacu *P. mesopotamicus* (*H*_*o*_ range from 0.068 to 0.911 and average of 8.5 alleles per locus), and tambaqui *C. macropomum* (*H*_*o*_ range from 0.430 to 0.880 and average of 12.8 alleles per locus; Calcagnotto and DeSalle, 2009; Fazzi-Gomes et al., 2017). In contrast to neutral markers (microsatellites in noncoding regions), gene-associated microsatellites might be more susceptible to selection pressure and, therefore, they have low values of gene diversity.

Analysis of the genetic diversity in pirapitinga farmed stocks showed significant differences between fish farms in different regions of Brazil, two from the Southeast (São Paulo State: SP1 and SP2) and two from the North (Tocantins State: TO1 and TO2). In general, farmed stocks were expected to have low genetic variability as a result of genetic decline, genetic drift, selection and inbreeding (Theodorou and Couvet, 2015). However, the results of this study showed higher genetic variability in breeding stocks from North fish farms in relation to the wild stocks (*p* < 0.05; higher values of allelic frequency and heterozygosity), which was also observed in studies with other related species (Barroso et al., 2005; Panarari-Antunes et al., 2011). The basis of this result could be considered from three different perspectives: (1) North fish farms had originated from different wild stocks resulting in high level of genetic variability; (2) problems of sample size bias, such as few microsatellite loci and individuals analyzed; (3) evidence for recent genetic bottlenecks in the wild population. Some studies of fish have reported bottlenecks in natural populations, particularly due to habitat loss and fragmentation by human disturbance (Brauer et al., 2016). In the case of pirapitinga, the fragmentation of the Tocantins River by hydroelectric dams in the 80′s and 90′s (e.g., Tucuruí and Luiz Eduardo Magalhães dams, where wild fish were collected for this study) could be responsible for a population reduction and subsequent genetic variation loss detected by our microsatellite analysis. There are considerable numbers of hydropower dams in the basin, which can affect the reproduction, migratory routes, and egg and larvae drift of fish (Agostinho et al., 2008). Alteration of the migratory flow consequently leads to a decrease in or interruption of the gene flow, reducing the population size, which makes the fish more susceptible to the effects of genetic drift (Hatanaka and Galetti, 2003), which results in genetic structure for some fish species (Calcagnotto and DeSalle, 2009; Do Prado et al., 2018).

STRUCTURE and pairwise *F*_ST_ analyses suggested a high genetic structure between the stocks herein analyzed, particularly as result of the fixation of specific alleles in some loci, which resulted in three clusters (Figure 3). There are three hypothetical explanations for these genetic patterns: (1) differentiation of wild population in relation to farmed stocks, which could be due to the selection of the fittest individuals for farming systems or low number of founders for the establishment of the farmed broodstocks; (2) lower genetic structure in North/wild than Southeast/wild, which suggests that North fish farms had frequent broodstock renovation from the wild; (3) fish farms were genetically clustered due to the geographic distribution, i.e., the degree of genetic similarity is higher when one fish farm is closer to the other, indicating interchange of individuals between nearby fish farms, common origin of the farmed broodstocks, or fixation/selection of specific alleles for different climatic conditions that are found in Brazil (North and South). However, these genetic patterns should be also evaluated using neutral markers (microsatellites in noncoding regions) and through techniques of higher genome coverage (SNP, single-nucleotide polymorphism).

Through AMOVA analysis, the main genetic variation was found to be present within populations (64.8%). This genetic pattern has also been reported in studies carried out with pacu (Calcagnotto and DeSalle, 2009; Iervolino et al., 2010) and tambaqui (Aguiar et al., 2013). Moreover, highly significant genetic variation was associated with differences between groups (Wild, SP, and TO), which represented 29.11% of genetic variation, in contrast to low differences among populations within groups (6.06%).

In general, our study of genetic characterization in piratininga farmed stocks provides important insights which can lead to better management of this species in aquaculture. Our results are fundamental to beginning a breeding programme, since the genetic structure should be taken into consideration when composing an initial base population, where matings between farmed individuals from North and Southeast Brazil are shown to result in higher genetic variability in the families. Moreover, the data suggested levels of genetic diversity which were higher in farmed stocks than in wild fish, discarding the occurrence of inbreeding. In general, lack of knowledge on genetic variability of stocks can result in inbreeding and fixation of deleterious genes, reduced growth rates, disease resistance problems and reduced ability to adapt to new environments (Arkush et al., 2002; Gallardo et al., 2004; Neira et al., 2006; Hillen et al., 2017). Therefore, besides the identification of QTL to assist in the selection of superior genotypes by MAS, studies of microsatellites are important for genetic monitoring, supporting pirapitinga aquaculture and increasing its productivity.

## Final considerations

The prospection of genetic data for pirapitinga is one of the priority issues for aquaculture, since this species is of high economic importance in national and global fish farming. The identification of gene-associated microsatellites by NGS is fundamental to understanding the genetic structure of wild and farmed populations, providing support for further management programmes and genetic pre-breeding programmes. Moreover, the microsatellites described herein are interesting targets used to find QTL markers, specifically related to the immune system of pirapitinga.

## Author contributions

PJ: Acquisition, analysis and interpretation of data, draft of the work, final approval of the version; VM-F, RA, and MdF: Draft of the work, development of intellectual content, final approval of the version; MH: Analysis and interpretation of data, draft of the work, final approval of the version; NM: Analysis and interpretation of data, draft of the work, final approval of the version; MV: Analysis and interpretation of data, draft of the work, final approval of the version; FP-F: Acquisition, Interpretation of data, development of intellectual content, final approval of the version; DH: Draft of the work, development of intellectual content, writing of the manuscript, final approval of the version.

### Conflict of interest statement

The authors declare that the research was conducted in the absence of any commercial or financial relationships that could be construed as a potential conflict of interest.
